# Effect of Equatorial Ligand Substitution on the Reactivity with Proteins of Paddlewheel Diruthenium Complexes: Structural Studies

**DOI:** 10.1021/acs.inorgchem.2c04103

**Published:** 2023-01-04

**Authors:** Aarón Terán, Giarita Ferraro, Ana E. Sánchez-Peláez, Santiago Herrero, Antonello Merlino

**Affiliations:** † Departamento de Química Inorgánica, Facultad de Ciencias Químicas, Universidad Complutense de Madrid, Madrid E-28040, Spain; ‡ Department of Chemical Sciences, University of Naples Federico II, Complesso Universitario di Monte Sant’Angelo via Cinthia 21, Naples 80126, Italy

## Abstract

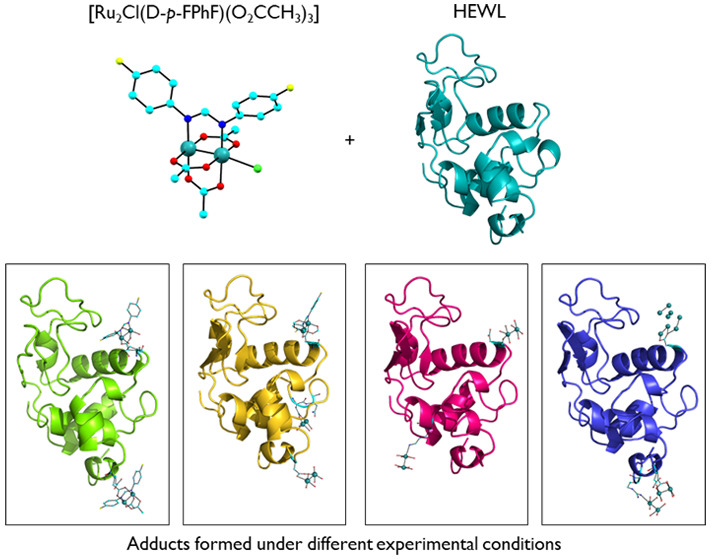

The paddlewheel [Ru_2_Cl(O_2_CCH_3_)_4_] complex was previously reported to react with the model protein hen egg white lysozyme (HEWL), forming adducts with two diruthenium moieties bound to Asp101 and Asp119 side chains upon the release of one acetate. To study the effect of the equatorial ligands on the reactivity with proteins of diruthenium compounds, X-ray structures of the adducts formed when HEWL reacts with [Ru_2_Cl(D-*p*-FPhF)(O_2_CCH_3_)_3_] [D-*p*-FPhF = *N*,*N*′-bis(4-fluorophenyl)formamidinate] under different conditions were solved. [Ru_2_Cl(D-*p*-FPhF)(O_2_CCH_3_)_3_] is bonded through their equatorial positions to the Asp side chains. Protein binding occurs cis or trans to D-*p*-FPhF. Lys or Arg side chains or even main-chain carbonyl groups can coordinate to the diruthenium core at the axial site. Data help to understand the reactivity of paddlewheel diruthenium complexes with proteins, providing useful information for the design of new artificial diruthenium-containing metalloenzymes with potential applications in the fields of catalysis, biomedicine, and biotechnology.

## Abstract 2

The molecular structures of the adducts formed when HEWL reacts with [Ru_2_Cl(D-p-FPhF)(O_2_CCH_3_)_3_], where D-*p*-FPhF is *N*,*N*′-bis(4-fluorophenyl)formamidinate, under four different conditions, were solved. Diruthenium centers coordinate Asp side chains at the equatorial site, losing one acetate ligand, Lys or Arg side chains, or even main-chain carbonyl groups at the axial site. Protein binding at Asp side chains can occur cis or trans to D-*p*-FPhF.

Most of the diruthenium complexes with the general formula [Ru_2_X(L–L)_4_] contain a strong metal–metal interaction (bond order of 2.5), four bridging equatorial ligands (L–L) arranged in a lantern-like fashion around the dimetallic center, and donor ligands (X) at the axial positions.^1−3^ These molecules have attracted considerable interest for their application in catalysis^4−10^ or biomedicine^11^ and for their peculiar magnetic and redox properties.^12−14^

The prototype of the diruthenium compounds family, [Ru_2_Cl(O_2_CCH_3_)_4_], has interesting pharmaceutical properties and has been used as a precursor to prepare promising anticancer compounds.^11,15−17^ For example, diruthenium compounds containing nonsteroidal antiinflammatory drugs^18−20^ or γ-linolenic acid^21,22^ as ligands have been found to be active against glioma tumor models *in vitro* and *in vivo* and, in particular, against human glioblastoma cell lines, while the [Ru_2_Cl(EB776)_4_] complex [where EB776 is the deprotonated form of (2-phenylindol-3-yl)glyoxyl-l-phenylalanine-l-leucine] was found to be active against a glioblastoma cell line.^23^ The diruthenium ibuprofenate complex also shows antiinflammatory properties with reduced gastric ulceration *in vivo* compared to the copper ibuprofenate complex.^24^

In earlier work, the interaction of [Ru_2_Cl(O_2_CCH_3_)_4_] with the model protein hen egg white lysozyme (HEWL) was studied to give the first key insights into the biological targets and mode of action of the diruthenium metallodrugs: the diruthenium center binds the protein, retaining the Ru–Ru bond and replacing one acetate ligand by an Asp side chain; a second acetate is then replaced by two water (H_2_O) molecules.^25^

Recently, it has been suggested that the use of bulky equatorial substituents on the diruthenium core may constitute an approach to increase the selectivity of diruthenium complexes toward anticancer targets.^26,27^ Many diruthenium complexes of the type [Ru_2_Cl(L–L)_4_] (L–L = *O,N* donors,^28,29^*N,N′*-donors^30,31^ or other *O,O′*-donors^3,32^) have been synthesized. However, intermediate substitution species [Ru_2_Cl(L–L)_*x*_(O_2_CCH_3_)_4–*x*_] (*x* = 1–3) are quite scarce.^14,33−35^ These molecules show variations in the stability, solubility, redox potential, and paramagnetic behavior compared to [Ru_2_Cl(O_2_CCH_3_)_4_] and to their fully substituted species.

Here, we investigate interaction of the monosubstituted diruthenium compound [Ru_2_Cl(D-*p*-FPhF)(O_2_CCH_3_)_3_], where D-*p*-FPhF = *N*,*N*′-bis(4-fluorophenyl)formamidinate (Figure 1), with HEWL under four different experimental conditions. [Ru_2_Cl(L–L)(O_2_CCH_3_)_3_] compounds are soluble in H_2_O and are expected to be more stable than [Ru_2_Cl(O_2_CCH_3_)_4_] according to the previous results.^34^

**Figure 1:**
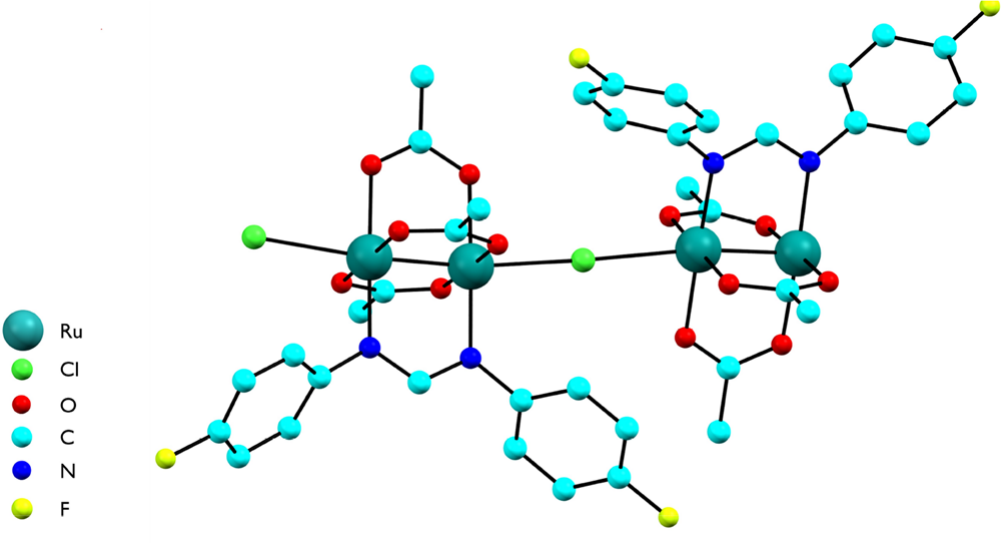
Asymmetric unit of [Ru_2_Cl(D-*p*-FPhF)(O_2_CCH_3_)_3_]_*n*_·2nCH_2_Cl_2_. Solvent molecules and H atoms have been omitted for clarity.

X-ray structures of adducts formed upon reaction of the protein with [Ru_2_Cl(D-*p*-FPhF)(O_2_CCH_3_)_3_] are reported. The results are compared with those obtained when proteins react with [Ru_2_Cl(O_2_CCH_3_)_4_]^25^ and various dirhodium compounds.^36−40^ The stability of [Ru_2_Cl(D-*p*-FPhF)(O_2_CCH_3_)_3_] was first studied in solution by UV–vis absorption spectroscopy (Figure 2). The spectrum in dichloromethane showed only one transition band around 480 nm.^34^ However, in H_2_O, the electronic spectrum shows bands in the UV (243 and 338 nm) and visible (∼420 and 520 nm) regions. A recent study by Kadish and co-workers^41^ with similar compounds showed the sensitivity of the axial positions to donor ligands [H_2_O or dimethyl sulfoxide (DMSO)] and suggested coordination to the axial positions.

**Figure 2:**
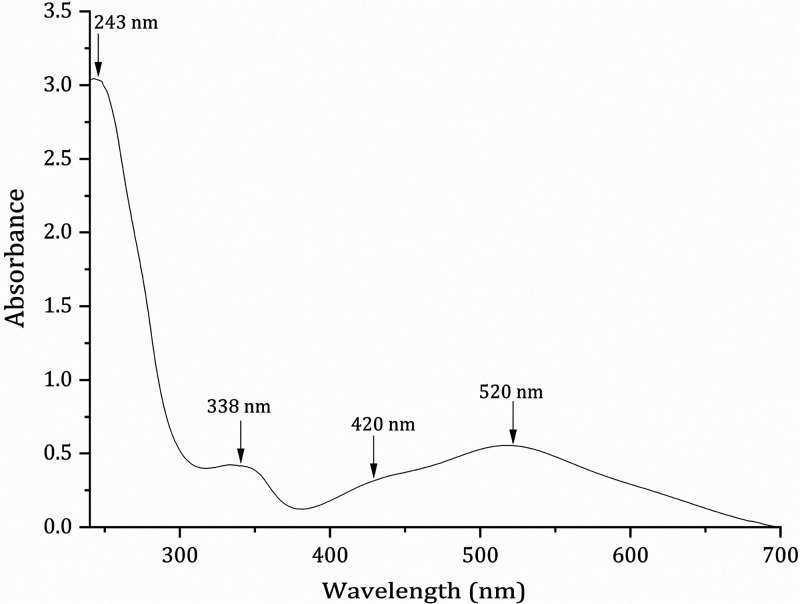
Time course UV–vis spectra of 50 μM [Ru_2_Cl(D-*p*-FPhF)(O_2_CCH_3_)_3_] in Milli-Q water. No appreciable spectral changes were observed within 24 h.

The higher-energy UV band is usually assigned to an axial ligand-to-metal charge transfer.^42^ The peak at 338 nm can be assigned to ligand-to-metal transitions [π(N) → σ*/π*/δ*(Ru_2_)], while the peaks in the visible region can be assigned to an allowed ligand-to-metal charge transfer [π(N), π(axial) → π*(Ru_2_)].^41^ These signals do not experience any change after 24 h.

UV–vis spectra of [Ru_2_Cl(D-*p*-FPhF)(O_2_CCH_3_)_3_] were also collected in the conditions that are used to grow HEWL crystals in the absence (Figures S1A–D) and presence of HEWL (protein to diruthenium compound molar ratio of 1:3; Figures S2A–D). In almost all of the conditions used for the crystallization experiments, ignorable variations in the spectral profiles of the compound are observed, while minimal variations are found in the presence of HEWL (Figures S1A–D and S2A–D). These findings suggest that the diruthenium compound is more stable than [Ru_2_Cl(O_2_CCH_3_)_4_] in different aqueous solutions^25^ and could bind the protein.

Circular dichroism spectra of the protein in the absence and presence of diruthenium are superimposable, with negligible variations of the molar ellipticity (Figure S3). These data suggest that HEWL retains its secondary structure and is presumably well-folded in the presence of [Ru_2_Cl(D-*p*-FPhF)(O_2_CCH_3_)_3_].

Fluorescence data confirm that the diruthenium compound binds the protein; indeed, a quenching of the intrinsic fluorescence of HEWL is observed when the metal compound concentration is increased (Figure S4A–F). The quenching is not accompanied by a change of the maximum emission wavelength, thus suggesting that metal compound binding occurs without the overall protein structure being altered.

Crystals of the adducts formed upon incubation of the protein with the metal compound were obtained by a soaking procedure under four different experimental conditions (see the Supporting Information for further details). The resolution of the structures ranges from 1.07 to 1.81 Å. Data collections and refinement statistics are reported in Table S1. The structures of the protein in the four adducts are very similar to each other and are not significantly affected by interaction with the metal compound (Figure 3). The root-mean-square deviation of Cα atoms from the structure of the metal-free protein (PDB code 193L)^43^ is within the range 0.17–0.21 Å.

**Figure 3:**
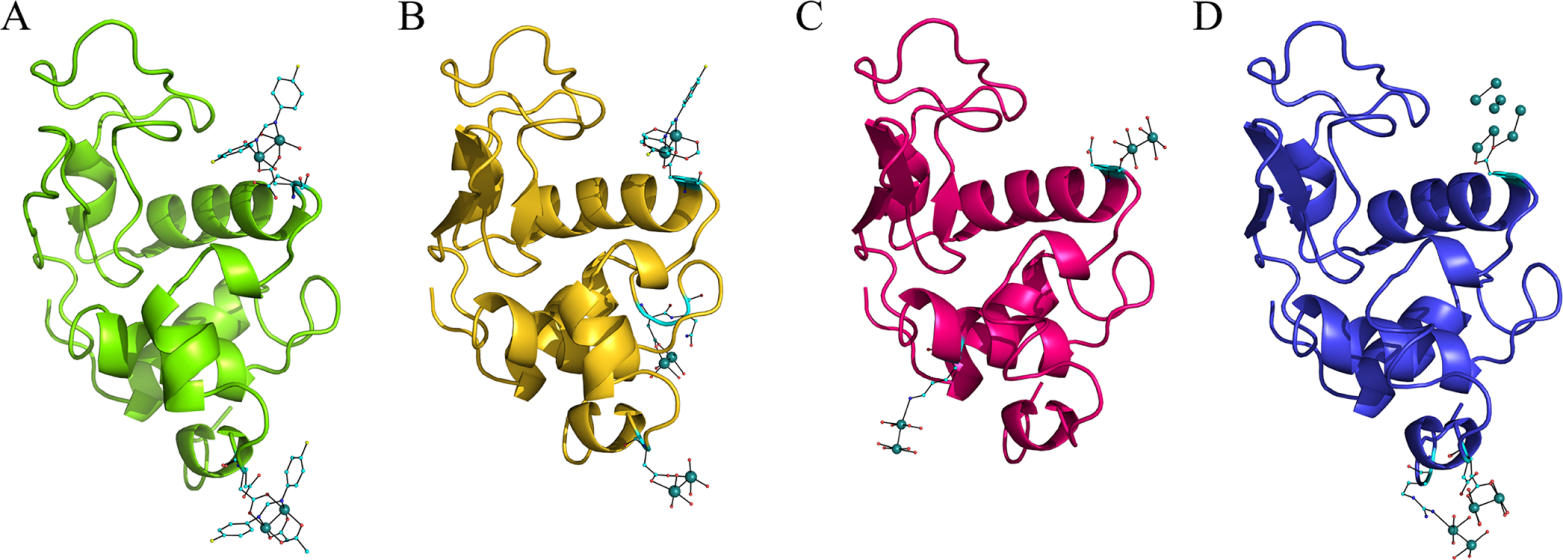
Overall structures of the adducts formed in the reaction of [Ru_2_Cl(D-*p*-FPhF)(O_2_CCH_3_)_3_] with HEWL under different experimental conditions: (A) structure **1** (20% ethylene glycol, 0.1 M sodium acetate buffer at pH 4.0, and 0.6 M sodium nitrate); (B) structure **2** (2.0 M sodium formate and 0.1 M HEPES buffer at pH 7.5); (C) structure **3** (0.8 M succinic acid at pH 7.0); (D) structure **4** (1.1 M sodium chloride and 0.1 M sodium acetate buffer at pH 4.0). Coordinates and structure factors are deposited in the PDB with codes 8BPH (structure **1**), 8BPU (structure **2**), 8BPJ (structure **3**), and 8BQM (structure **4**). Ru atoms are in green.

In structure **1** (Figure 3A), diruthenium centers are found to be close to the side chains of Asp101 or Asp119 (Figures 4A,B). The diruthenium-containing fragment bound close to Asp119 is well-defined (Figure 4B), while the D-*p*-FPhF ligand of the diruthenium bound to Asp101 is less ordered (Figure 4A). The two diruthenium-containing fragments are alternative to each other with 0.50 and 0.40 occupancies, respectively. The Asp119 side chain changes its conformation to coordinate the diruthenium center at the equatorial site (Figure S5), while Asp101 is already in the right position in the metal-free protein to link the dimetallic center. Close to the Asp119 side chain, the diruthenium unit binds two acetate ligands along with one L–L bridging ligand and the carboxylate group of Asp at the equatorial position. Close to the Asp101 side chain, acetate ligands could be replaced by H_2_O molecules. Because of the crystallization conditions (low pH and high concentration of sodium nitrate), we cannot exclude that, in structure **1**, acetate ligands were replaced by nitrate ions. The axial positions of the diruthenium center bound to Asp119 are occupied by H_2_O molecules, while at the Asp101 binding site, axial ligands were not added to the model because of low electron density. Notably, close to Asp119, the diruthenium-containing fragment binds the Asp side chain cis to the D-*p*-FPhF ligand, while close to Asp101, the side chain is trans to the L–L ligand. As was already observed, bisubstituted species usually have cis configuration. The trans disposition has only been obtained with bulky equatorial ligands.^14^

**Figure 4:**
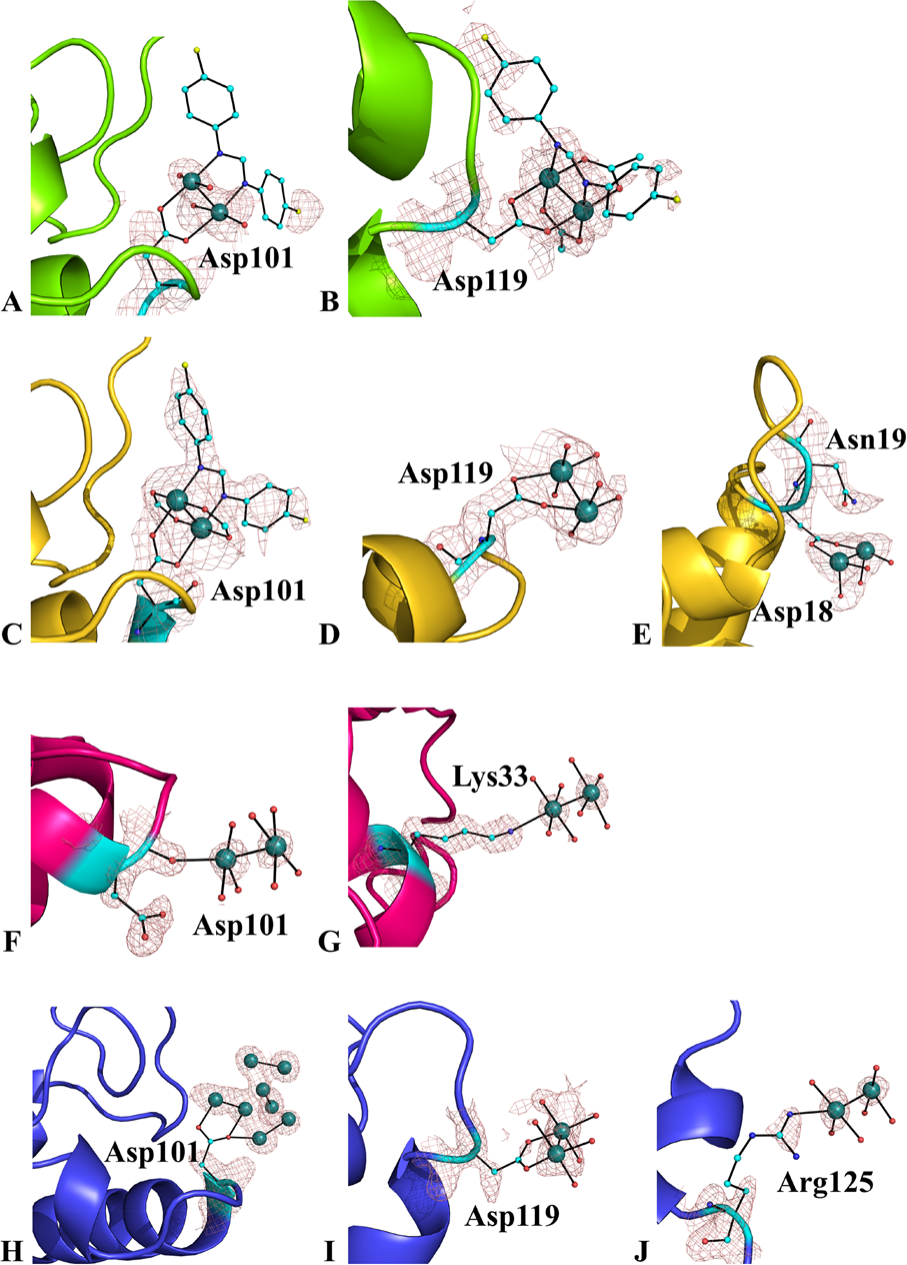
Diruthenium binding sites in the adducts formed upon the reaction of HEWL with [Ru_2_Cl(D-*p*-FPhF)(O_2_CCH_3_)_3_] in structures **1** (panels A and B), **2** (panels C–E), **3** (panels F and G), and **4** (panels H–J). The electron density maps are very well-defined and unambiguously indicate that the compound retains the diruthenium center and L–L ligands upon protein binding. Axial H_2_O molecules are omitted for the sake of clarity. 2*F*_o_ – *F*_c_ electron density maps are contoured at 1.0 σ (salmon).

In structure **2** (Figure 3B), similar results were obtained (Figure 4C–E): the diruthenium center binds Asp101, with the side chain trans to the L–L ligand (occupancy = 0.70; Figure 4C) and to Asp119 (occupancy = 0.55; Figure 4D). Interestingly, in this structure, the diruthenium bound to Asp101 with the side chain trans to the D-*p*-FPhF ligand (Figure 4C) is better defined than that bound to Asp119 (Figure 4D). In the former diruthenium binding site, inspection of the electron density maps suggests that two formate ions have replaced acetate ligands (Figure 4C). Axially coordinated H_2_O molecules can also be confidently modeled. In the latter, the electron density is disordered and diruthenium ligands have been interpreted as H_2_O molecules (Figure 4D). An additional diruthenium binding site with low occupancy (0.20) is found to be close to the side chain of Asp18 (Figure 4E). Here, only two Ru atoms and a few H_2_O molecules as ligands have been modeled. The Ru atoms are at ∼3 Å from the atoms of the Asn19 side chain.

Additional binding sites for the diruthenium core were found also in structure **3** (Figure 3C). In this structure, a diruthenium center is axially coordinated to the carbonyl of Asp101 (Figure 4F) and to the Lys33 side chain (Figure 4G). Unfortunately, in both of these additional diruthenium binding sites, metal ligands cannot be confidently modeled because of the low occupancy of the metal (0.25 and 0.20, respectively) and conformational disorder. The carbonyl group probably competes with H_2_O molecules in solution. Nevertheless, the axial coordination of dimetallic compounds to both a residue side chain and a carbonyl oxygen in the solid state was already observed in the adducts formed upon the reaction of dirhodium compounds with proteins.^36−40,44^

In structure **4** (Figure 3D), binding of the dimetallic center occurs at the level of the side chains of Asp101 (Figure 4H), Asp119 (Figure 4I), and Arg125 (Figure 4J). However, in this structure, especially close to the side chain of Asp101 (Figure 4H), interpretation of the map is complicated by conformational disorder, by the presence of multiple conformations of the diruthenium center, and by the presence of a high concentration of chloride ions, which could replace the diruthenium ligands.^45^ Interestingly, the diruthenium center close to the side chain of Arg125 is axially coordinated (Figure 4J).

Overall, these data indicate that Ru–Ru bonds remain stable upon reaction with HEWL regardless of the experimental conditions used. Interestingly, the structures of the adducts of HEWL with dirhodium tetraacetate and derivatives under the same conditions show breakage of the Rh–Rh bond.^36,38,39^

In conclusion, here we have studied the reactivity of [Ru_2_Cl(D-*p*-FPhF)(O_2_CCH_3_)_3_] with HEWL under different experimental conditions. Our data unambiguously demonstrate that the compound binds the protein, forming adducts with dimetallic moieties bound to the Asp side chains upon the release of an acetate ligand. In the adduct, excluding the acetate replaced by the Asp side chain, the other ligands can be retained and the D-*p*-FPhF ligand can be cis or trans to the Asp side chain probably due to steric hindrance. These data confirm that diruthenium compounds react with proteins, forming adducts with Asp side chains at equatorial sites^24^ and keeping their paddlewheel structure. The results also indicate that monosubstituted diruthenium compounds present a different reactivity with proteins compared to diruthenium tetraacetate^25^ and to paddlewheel dirhodium compounds.^36−40,44^

Our data also suggest the possibility of an axial bond of the diruthenium core to the side chains of Lys or Arg and to backbone carbonyl groups. It is possible that the binding of the Asp residues at the equatorial position is a late event in the reaction of paddlewheel complexes with proteins and that the coordination of protein nucleophile sites at the axial position of the bimetallic scaffolds can be an early event in the dimetallic compound/protein recognition process. The binding to the axial site not only anticipates the later acetate detachment but also could exert a structural destabilization that facilitates its eventual occurrence, as indicated by computational studies.^26^ The combination of axial and equatorial coordinative binding has been postulated as a way to establish specific interactions between [Ru_2_Cl_2_(formamidinate)_3_(DMSO)] and ribonucleic acid.^46^

## Supporting Information Available

Materials, solution studies, crystallization and X-ray structure solution and refinement, Figures S1–S5, and Table S1 (PDF)
