# Supplementary material for: Effect of Equatorial Ligand Substitution on the Reactivity with Proteins of Paddlewheel Diruthenium Complexes: Structural Studies
Source: Inorg Chem. 2023 Jan 4;62(2):670–4. doi: 10.1021/acs.inorgchem.2c04103 (PMC9846696; doi:10.1021/acs.inorgchem.2c04103)
Supplement: Supplementary file 1 — ic2c04103_si_001.pdf [file ic2c04103_si_001.pdf]

Supporting Information

Effect of equatorial ligand substitution on the reactivity with proteins of paddlewheel diruthenium complexes: structural studies

Aarón Terán,[a] Giarita Ferraro,[b] Ana E. Sánchez-Peláez,[a] Santiago Herrero[a]* and Antonello Merlino[b]*

[a]Departamento de Química Inorgánica, Facultad de Ciencias Químicas, Universidad Complutense de Madrid, E-28040 Madrid, Spain.

[b]Department of Chemical Sciences, University of Naples Federico II, Complesso Universitario di Monte Sant'Angelo, via Cinthia, 21, 80126, Naples, Italy.

*E-mails: antonello.merlino@unina.it or sherrero@ucm.es

Index

Materials 3

In solution studies 3

Crystallization and X-ray structure solution and refinement 4

Figure S1. Time course UV–vis spectra of 50 μM [Ru_2Cl(D-*p*-FPhF)(μ-O_2CCH_3)$_3$] in: (**A**) 20% ethylene glycol, 0.1 M sodium acetate buffer at pH 4.0, 0.6 M sodium nitrate; (**B**) 2.0 M sodium formate, 0.1 M Hepes buffer pH 7.5; (**C**) 0.8 M succinic acid pH 7.0; (**D**) 1.1 M sodium chloride, 0.1 M sodium acetate buffer pH 4.0......... 5

Figure S2. Time course UV–vis spectra of 50 μM [Ru_2Cl(D-*p*-FPhF)(μ-O_2CCH_3)$_3$] in the presence of HEWL (protein to diruthenium molar ratio= 1:3) in: (**A**) 20% ethylene glycol, 0.1 M sodium acetate buffer at pH 4.0, 0.6 M sodium nitrate; (**B**) 2.0 M sodium formate, 0.1 M Hepes buffer pH 7.5; (**C**) 0.8 M succinic acid pH 7.0; (**D**) 1.1 M sodium chloride, 0.1 M sodium acetate buffer pH 4.0. 6

Figure S3. Far-UV CD spectra of HEWL (7.0 μM concentration) incubated for 1 h in the presence of [Ru_2Cl(D-*p*-FPhF)(μ-O_2CCH_3)$_3$] in 10 mM sodium acetate buffer pH 4.0 at protein to diruthenium molar ratio = 1:1 (violet) , 1:2 (red) and 1:3 (pink). CD of metal-free protein is in black. 7

Figure S4. Fluorescence emission spectra of HEWL in (**A-B**) 10 mM sodium acetate buffer pH 4.0, (**C-D**) 10 mM Hepes buffer pH 7.5 and (**E-F**) 10 mM succinic acid pH 7.0 upon titration with a solution of [Ru_2Cl(D-*p*-FPhF)(μ-O_2CCH_3)$_3$]. Spectra have been collected using λ_{exc} = 280 nm (panels **A**, **C** and **E**) and 295 nm (panels **B**, **D** and **F**). 8

Figure S5. Conformation of Asp119 side chain in the metal-free HEWL (panel A, violet) and in structure **2** (panel B, yellow). 9

Table S1. Data collection and refinement statistics. 10

Materials

HEWL was purchased from Sigma Chemical Co (Merck Life Science S.r.l., Milan, Italy) at highest grade of purity and used without further purification. Synthesis of $[Ru_2Cl(D\text{-}p\text{-}FPhF)(O_2CCH_3)_3]$ was carried out as done in a previous work[1] using the following procedure.

A round-bottom flask containing 0.142 g (0.30 mmol) of $[Ru_2Cl(O_2CCH_3)_4]$ starting material, 0.070 g (0.30 mmol) of HD-*p*-FPhF ligand, 0.1 mL (0.72 mmol) of Et_3N and 50 mL of ethanol was sonicated for 2 h at 80 kHz. The mixture was filtered through Celite® to remove unreacted $[Ru_2Cl(O_2CCH_3)_4]$, and the solution was evaporated under vacuum. The solid was washed with diethyl ether (50 mL), extracted in distilled water (150 mL), and filtered through Celite®. The filtered solution was washed with 5 mL of brine and extracted with dichloromethane (2 × 50 mL). The organic solution was treated with $MgSO_4$, filtered off, evaporated under vacuum giving rise to a red-wine solid. Yield: 150 mg (75%). Anal. found (calculated) for $[Ru_2Cl(N_2F_2C_{13}H_9)(O_2C_2H_3)_3(OH_2)]$ (663.97 g□mol^{-1}): C, 34.49 (34.37); H, 3.03 (3.04); N, 4.41 (4.22). MS (ESI$^+$) m/z: 610 $[M - Cl - H_2O]^+$ (55%); 690 $[M + CH_3CO]^+$ (100%). IR signals: 3380w (νOH), 3056w (νCH_{ar}), 2936w (νCH), 1639m (δHOH), 1602w (νCC_{ar}), 1526w, 1497s, ($\nu CN + \nu CC_{ar}$), 1434s (νCOO_a), 1411m (δCH), 1351m (νCOO_s), 1311m 1201s (νCN), 1153s (νCF), 1095m ($\delta C_{ar}C_{ar}H$), 1014m (δCH), 944m (δCH), 865w (δNCH), 834s ($\delta C_{ar}C_{ar}H$), 797m ($\delta C_{ar}C_{ar}H$), and 687s (δCOO) cm^{-1}. μ_{eff} (25 °C) = 4.0 μ_B.

In solution studies

UV-vis spectra of $[Ru_2Cl(D\text{-}p\text{-}FPhF)(O_2CCH_3)_3]$ were recorded on a Jasco V-750 spectrophotometer using quartz cuvette of 1 cm path length, a diruthenium concentration of 50 µM in water and in other four different experimental conditions, i.e. those used to grow HEWL crystals: A) 20% ethylene glycol, 0.1 M sodium acetate buffer at pH 4.0, 0.6 M sodium nitrate; B) 2.0 M sodium formate, 0.1 M Hepes buffer pH 7.5; C) 0.8 M succinic acid pH 7.0; D) 1.1 M sodium chloride, 0.1 M sodium acetate buffer pH 4.0. Spectra were collected for 5 hours continuously, and then after 24 hours and 7 days, in the absence and in the presence of HEWL (protein to metal compound molar ratio 1:3). Other experimental parameters were: wavelength range 240 – 700 nm, data pitch 1.0 nm, scanning speed 400 nm/min, band width 2.0 nm. Each measurement was repeated twice.

Far UV-CD spectra were recorded on a Jasco J-715 spectropolarimeter equipped with a Peltier thermostatic cell holder (Model PTC-348WI) in the range of 190-250 nm, using a protein concentration of 7 µM and a quartz cell with 0.1 cm path length. HEWL was incubated 1 h with an increasing amounts of $[Ru_2Cl(D\text{-}p\text{-}FPhF)(O_2CCH_3)_3]$. Protein to diruthenium molar ratios were: 1:1, 1:2 and 1:3. Spectra were collected in: 10 mM sodium acetate buffer at pH 4.0. Other experimental parameters were 1.0 nm data pitch, 2.0 nm bandwidth, 50 nm/min scanning speed, 2.0 s response time, and 25 °C. Each spectrum was obtained by averaging three scans.

A HORIBA Fluoromax-4 spectrofluorometer equipped with a thermostat bath and a 1 cm path length cuvette was used to register fluorescence spectra of HEWL in the absence and in the presence of $[Ru_2Cl(D\text{-}p\text{-}FPhF)(O_2CCH_3)_3]$ at 25°C. HEWL solutions at a concentration of 1.4 µM were titrated with a 2 mM Ru_2 compound solution. The protein was excited at 280 nm (to follow Tyr and Trp emission) and 295 nm (to follow Trp emission only) over a range of wavelengths between 295 and 450 nm and between 310 and 450 nm, for excitation at 280 and 295 nm, respectively. Different protein to metal molar ratios were reached upon titration: 1:0.2, 1:0.5, 1:1, 1:2, 1:4, 1:6, 1:8, 1:10. Solutions were stirred and equilibrated for 5 minutes before recording the spectra. The titrations were carried out in three different buffers: 10 mM sodium acetate buffer pH 4.4, 10 mM Hepes buffer pH 7.5 and 10 mM succinic acid pH 7.0. When necessary, correction of the fluorescence spectra was employed to compensate the effects of the existing primary and/or secondary internal filter using the following equation:

$$F_{corr} = F_{obs}\, 10^{\,(Aex + Aem) / 2}$$

where F_{corr} and F_{obs} are, respectively, the corrected and observed fluorescence intensities, while A_{ex} and A_{em} are the absorbance values, respectively, at the excitation and emission wavelengths.

Each measurement was repeated twice.

Crystallization and X-ray structure solution and refinement

Crystals of HEWL were grown by the hanging drop vapor diffusion method mixing 1 µL of protein solution (concentration 13 mg/mL) with an equal volume of the reservoir solution containing the following conditions: A) 20% ethylene glycol, 0.1 M sodium acetate at pH 4.0, and 0.6 M sodium nitrate, B) 2.0 M sodium formate, 0.1 M Hepes buffer pH 7.5; C) 0.8 M succinic acid pH 7.0. D) 1.1 M sodium chloride, 0.1 M sodium acetate buffer pH 4.0. Crystals grew in few days. These crystals were then exposed to stabilizing solutions containing the mother liquors and a saturated solution of $[Ru_2Cl(D\text{-}p\text{-}FPhF)(O_2CCH_3)_3]$ for a soaking time of two weeks.

Crystals of the adducts of $[Ru_2Cl(D\text{-}p\text{-}FPhF)(O_2CCH_3)_3]$ with HEWL obtained under different conditions diffract X-ray in the resolution range of 1.17-1.81 Å. X-ray diffraction data collections were carried out on Beamline XRD2 at Elettra synchrotron (Trieste, Italy) [2], using a wavelength of 1.00 Å and a cold nitrogen stream of 100 K. Before exposure to X-ray, crystals were cryoprotected using a solution of the reservoir with 25% glycerol. Data processing and scaling were performed using a Global Phasing autoPROC pipeline[3]. Data collection statistics are reported in Table S1.

The structures were solved by molecular-replacement method using Phaser[4] with PDB entry 193L[5] as template. Refmac[6] was used for the refinement and Coot[7] for manual model building. The Ru atom positions were validated using anomalous difference electron density maps. Ligand positions were restraints to guide geometry optimization. Pymol (www.pymol.org) was used to generate molecular graphic figures. Coordinates and structure factors of the adducts were deposited in the Protein Data Bank under the accession codes **8BPH** (structure **1**), **8BPU** (structure **2**), **8BPJ** (structure **3**), **8BQM** (structure **4**).

References

1. Inchausti, A.; Terán, A.; Manchado-Parra, A.; de Marcos Galán, A.; Perles, J.; Cortijo, M.; González-Prieto, R.; Herrero, S.; Jiménez-Aparicio, R. New insights into progressive ligand replacement from $[Ru_2Cl(O_2CCH_3)_4]$: synthetic strategies and variation in redox potentials and paramagnetic shifts. *Dalton Trans*. **2022**, *51*, 9708-9719.

2 Lausi, A.; Polentarutti, M.; Onesti, S.; Plaisier, J. R.; Busetto, E.; Bais, G.; Barba, L.; Cassetta, A.; Campi, G.; Lamba, D.; Pifferi, A.; Mande, S. C.; Sarma, D. D.; Sharma, S. M.; Paolucci, G. Status of the crystallography beamlines at Elettra. *Eur. Phys. J. Plus*, **2015**, 130, 1–8.

3. Vonrhein C.; Flensburg C.; Keller P.; Sharff A.; Smart O.; Paciorek W.; Womack T.; Bricogne G. Data processing and analysis with the autoPROC toolbox. *Acta Crystallogr., Sect. D: Biol. Crystallogr.* **2011**, *67*, 293-302.

4. McCoy A. J.; Grosse-Kunstleve R. W.; Adams P. D.; Winn M. D.; Storoni L. C.; Read R. J. Phaser crystallographic software. *J. Appl. Crystallogr.* **2007**, *40*, 658–674.

5. Vaney M. C.; Maignan S.; Riès-Kautt M.; Ducruix A. High-Resolution Structure (1.33 Å) of a HEW Lysozyme Tetragonal Crystal Grown in the APCF Apparatus. Data and Structural Comparison with a Crystal Grown under Microgravity from SpaceHab-01 Mission. *Acta Crystallogr., Sect. D: Biol. Crystallogr.* **1996**, *52*, 505-517.

6. Murshudov G.N.; Vagin A.A; Dodson E.J. Refinement of Macromolecular Structures by the Maximum-Likelihood method". *Acta Crystallogr., Sect. D: Biol. Crystallogr.* **1997**, *53*, 240-255.

7. Emsley P.; Lohkamp B.; Scott W. G.; Cowtan K. Features and development of *Coot*. *Acta Crystallogr., Sect. D: Biol. Crystallogr.* **2010**, *66*, 486–501.

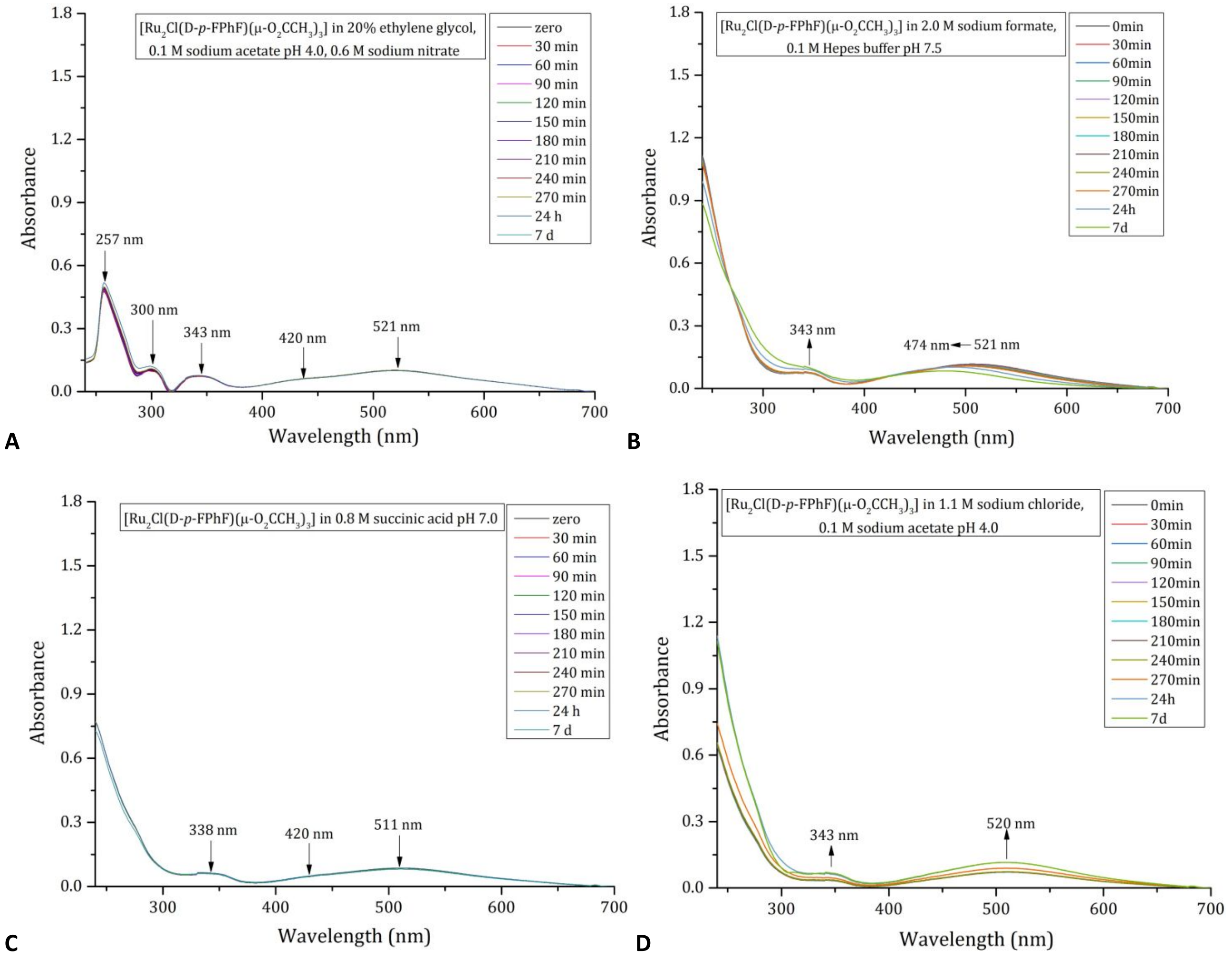

Figure S1. Time course UV–vis spectra of 50 μM [Ru_2Cl(D-*p*-FPhF)(O_2CCH_3)$_3$] in: (**A**) 20% ethylene glycol, 0.1 M sodium acetate buffer at pH 4.0, 0.6 M sodium nitrate; (**B**) 2.0 M sodium formate, 0.1 M Hepes buffer pH 7.5; (**C**) 0.8 M succinic acid pH 7.0; (**D**) 1.1 M sodium chloride, 0.1 M sodium acetate buffer pH 4.0.

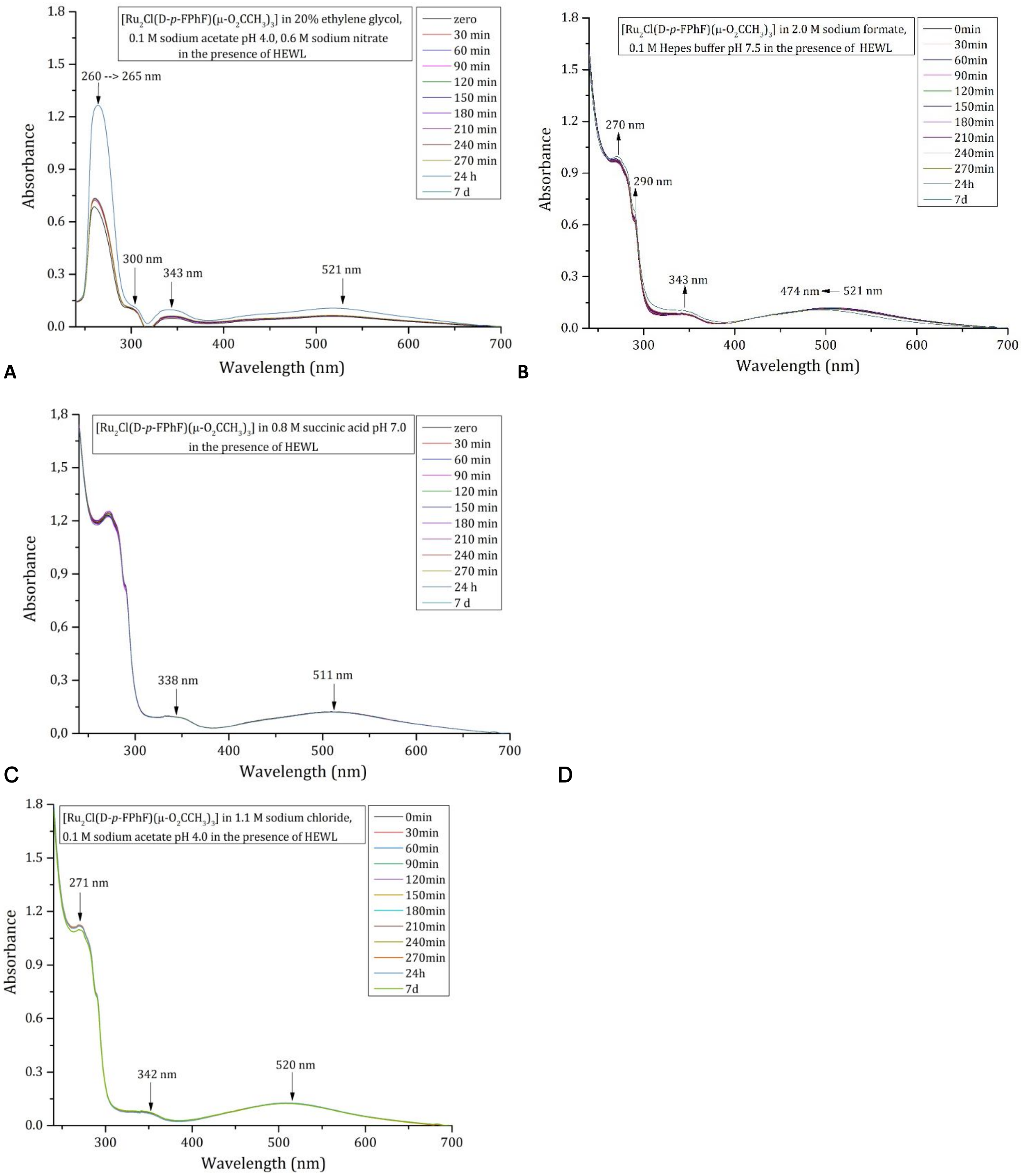

Figure S2. Time course UV–vis spectra of 50 μM $[Ru_2Cl(D\text{-}p\text{-}FPhF)(O_2CCH_3)_3]$ in the presence of HEWL (protein to diruthenium molar ratio = 1:3) in: (**A**) 20% ethylene glycol, 0.1 M sodium acetate buffer at pH 4.0, 0.6 M sodium nitrate; (**B**) 2.0 M sodium formate, 0.1 M Hepes buffer pH 7.5; (**C**) 0.8 M succinic acid pH 7.0; (**D**) 1.1 M sodium chloride, 0.1 M sodium acetate buffer pH 4.0.

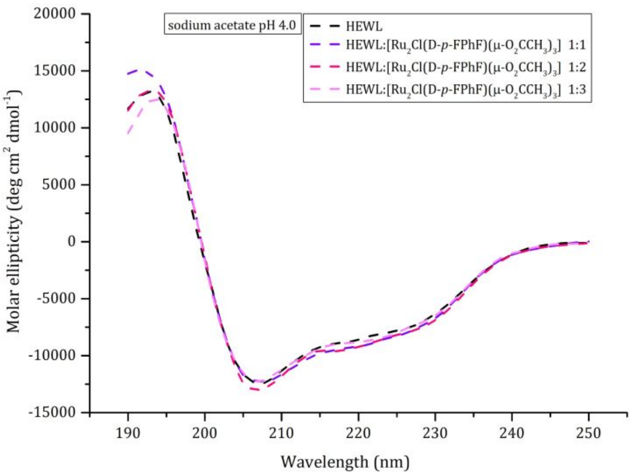

Figure S3. Far-UV CD spectra of HEWL (7.0 μM concentration) incubated for 1 h in the presence of [Ru_2Cl(D-*p*-FPhF)(O_2CCH_3)$_3$] in 10 mM sodium acetate buffer pH 4.0 at protein to diruthenium molar ratio = 1:1 (violet) , 1:2 (red) and 1:3 (pink). CD of metal-free protein is in black.

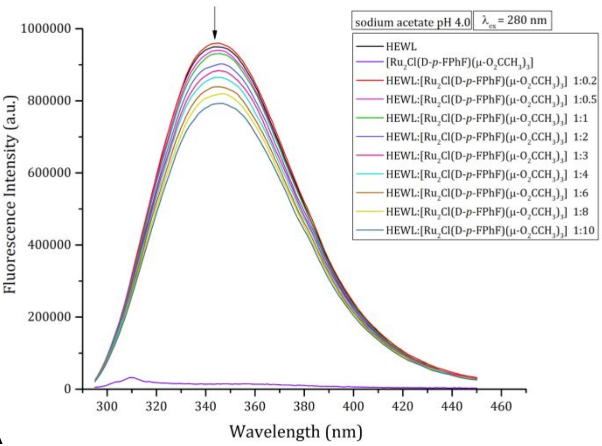
sodium acetate pH 4.0
λ_{ex} = 280 nm
HEWL
$[Ru_2Cl(D\text{-}p\text{-}FPhF)(\mu\text{-}O_2CCH_3)_3]$
HEWL:$[Ru_2Cl(D\text{-}p\text{-}FPhF)(\mu\text{-}O_2CCH_3)_3]$ 1:0.2
HEWL:$[Ru_2Cl(D\text{-}p\text{-}FPhF)(\mu\text{-}O_2CCH_3)_3]$ 1:0.5
HEWL:$[Ru_2Cl(D\text{-}p\text{-}FPhF)(\mu\text{-}O_2CCH_3)_3]$ 1:1
HEWL:$[Ru_2Cl(D\text{-}p\text{-}FPhF)(\mu\text{-}O_2CCH_3)_3]$ 1:2
HEWL:$[Ru_2Cl(D\text{-}p\text{-}FPhF)(\mu\text{-}O_2CCH_3)_3]$ 1:3
HEWL:$[Ru_2Cl(D\text{-}p\text{-}FPhF)(\mu\text{-}O_2CCH_3)_3]$ 1:4
HEWL:$[Ru_2Cl(D\text{-}p\text{-}FPhF)(\mu\text{-}O_2CCH_3)_3]$ 1:6
HEWL:$[Ru_2Cl(D\text{-}p\text{-}FPhF)(\mu\text{-}O_2CCH_3)_3]$ 1:8
HEWL:$[Ru_2Cl(D\text{-}p\text{-}FPhF)(\mu\text{-}O_2CCH_3)_3]$ 1:10
Fluorescence Intensity (a.u.)
Wavelength (nm)

A

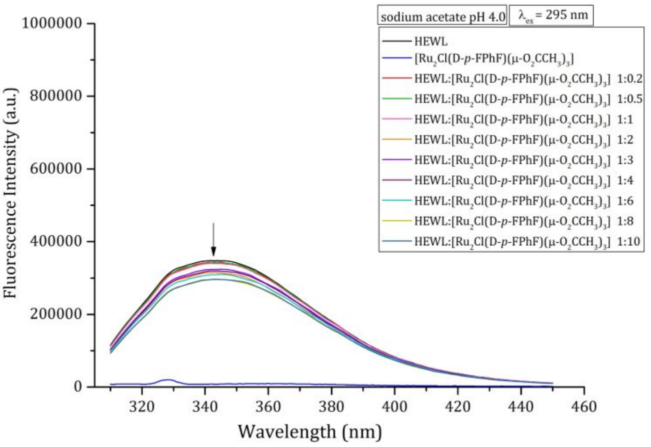
sodium acetate pH 4.0
λ_{ex} = 295 nm
HEWL
$[Ru_2Cl(D\text{-}p\text{-}FPhF)(\mu\text{-}O_2CCH_3)_3]$
HEWL:$[Ru_2Cl(D\text{-}p\text{-}FPhF)(\mu\text{-}O_2CCH_3)_3]$ 1:0.2
HEWL:$[Ru_2Cl(D\text{-}p\text{-}FPhF)(\mu\text{-}O_2CCH_3)_3]$ 1:0.5
HEWL:$[Ru_2Cl(D\text{-}p\text{-}FPhF)(\mu\text{-}O_2CCH_3)_3]$ 1:1
HEWL:$[Ru_2Cl(D\text{-}p\text{-}FPhF)(\mu\text{-}O_2CCH_3)_3]$ 1:2
HEWL:$[Ru_2Cl(D\text{-}p\text{-}FPhF)(\mu\text{-}O_2CCH_3)_3]$ 1:3
HEWL:$[Ru_2Cl(D\text{-}p\text{-}FPhF)(\mu\text{-}O_2CCH_3)_3]$ 1:4
HEWL:$[Ru_2Cl(D\text{-}p\text{-}FPhF)(\mu\text{-}O_2CCH_3)_3]$ 1:6
HEWL:$[Ru_2Cl(D\text{-}p\text{-}FPhF)(\mu\text{-}O_2CCH_3)_3]$ 1:8
HEWL:$[Ru_2Cl(D\text{-}p\text{-}FPhF)(\mu\text{-}O_2CCH_3)_3]$ 1:10
Fluorescence Intensity (a.u.)
Wavelength (nm)

B

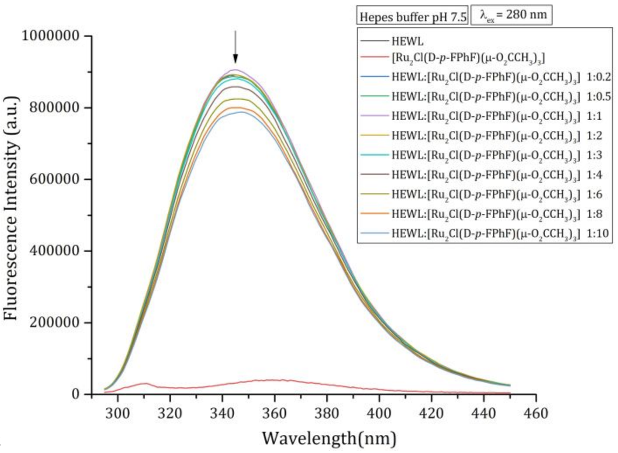
Hepes buffer pH 7.5
λ_{ex} = 280 nm
HEWL
$[Ru_2Cl(D\text{-}p\text{-}FPhF)(\mu\text{-}O_2CCH_3)_3]$
HEWL:$[Ru_2Cl(D\text{-}p\text{-}FPhF)(\mu\text{-}O_2CCH_3)_3]$ 1:0.2
HEWL:$[Ru_2Cl(D\text{-}p\text{-}FPhF)(\mu\text{-}O_2CCH_3)_3]$ 1:0.5
HEWL:$[Ru_2Cl(D\text{-}p\text{-}FPhF)(\mu\text{-}O_2CCH_3)_3]$ 1:1
HEWL:$[Ru_2Cl(D\text{-}p\text{-}FPhF)(\mu\text{-}O_2CCH_3)_3]$ 1:2
HEWL:$[Ru_2Cl(D\text{-}p\text{-}FPhF)(\mu\text{-}O_2CCH_3)_3]$ 1:3
HEWL:$[Ru_2Cl(D\text{-}p\text{-}FPhF)(\mu\text{-}O_2CCH_3)_3]$ 1:4
HEWL:$[Ru_2Cl(D\text{-}p\text{-}FPhF)(\mu\text{-}O_2CCH_3)_3]$ 1:6
HEWL:$[Ru_2Cl(D\text{-}p\text{-}FPhF)(\mu\text{-}O_2CCH_3)_3]$ 1:8
HEWL:$[Ru_2Cl(D\text{-}p\text{-}FPhF)(\mu\text{-}O_2CCH_3)_3]$ 1:10
Fluorescence Intensity (a.u.)
Wavelength(nm)

C

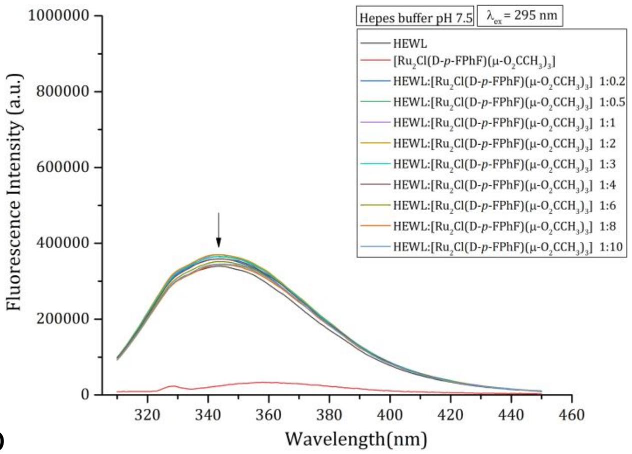
Hepes buffer pH 7.5
λ_{ex} = 295 nm
HEWL
$[Ru_2Cl(D\text{-}p\text{-}FPhF)(\mu\text{-}O_2CCH_3)_3]$
HEWL:$[Ru_2Cl(D\text{-}p\text{-}FPhF)(\mu\text{-}O_2CCH_3)_3]$ 1:0.2
HEWL:$[Ru_2Cl(D\text{-}p\text{-}FPhF)(\mu\text{-}O_2CCH_3)_3]$ 1:0.5
HEWL:$[Ru_2Cl(D\text{-}p\text{-}FPhF)(\mu\text{-}O_2CCH_3)_3]$ 1:1
HEWL:$[Ru_2Cl(D\text{-}p\text{-}FPhF)(\mu\text{-}O_2CCH_3)_3]$ 1:2
HEWL:$[Ru_2Cl(D\text{-}p\text{-}FPhF)(\mu\text{-}O_2CCH_3)_3]$ 1:3
HEWL:$[Ru_2Cl(D\text{-}p\text{-}FPhF)(\mu\text{-}O_2CCH_3)_3]$ 1:4
HEWL:$[Ru_2Cl(D\text{-}p\text{-}FPhF)(\mu\text{-}O_2CCH_3)_3]$ 1:6
HEWL:$[Ru_2Cl(D\text{-}p\text{-}FPhF)(\mu\text{-}O_2CCH_3)_3]$ 1:8
HEWL:$[Ru_2Cl(D\text{-}p\text{-}FPhF)(\mu\text{-}O_2CCH_3)_3]$ 1:10
Fluorescence Intensity (a.u.)
Wavelength(nm)

D

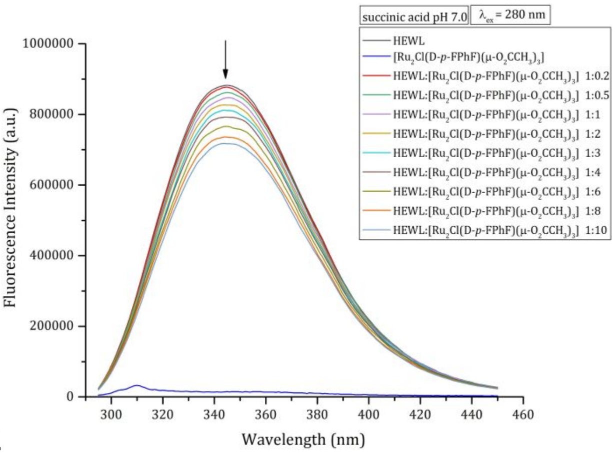
succinic acid pH 7.0
λ_{ex} = 280 nm
HEWL
$[Ru_2Cl(D\text{-}p\text{-}FPhF)(\mu\text{-}O_2CCH_3)_3]$
HEWL:$[Ru_2Cl(D\text{-}p\text{-}FPhF)(\mu\text{-}O_2CCH_3)_3]$ 1:0.2
HEWL:$[Ru_2Cl(D\text{-}p\text{-}FPhF)(\mu\text{-}O_2CCH_3)_3]$ 1:0.5
HEWL:$[Ru_2Cl(D\text{-}p\text{-}FPhF)(\mu\text{-}O_2CCH_3)_3]$ 1:1
HEWL:$[Ru_2Cl(D\text{-}p\text{-}FPhF)(\mu\text{-}O_2CCH_3)_3]$ 1:2
HEWL:$[Ru_2Cl(D\text{-}p\text{-}FPhF)(\mu\text{-}O_2CCH_3)_3]$ 1:3
HEWL:$[Ru_2Cl(D\text{-}p\text{-}FPhF)(\mu\text{-}O_2CCH_3)_3]$ 1:4
HEWL:$[Ru_2Cl(D\text{-}p\text{-}FPhF)(\mu\text{-}O_2CCH_3)_3]$ 1:6
HEWL:$[Ru_2Cl(D\text{-}p\text{-}FPhF)(\mu\text{-}O_2CCH_3)_3]$ 1:8
HEWL:$[Ru_2Cl(D\text{-}p\text{-}FPhF)(\mu\text{-}O_2CCH_3)_3]$ 1:10
Fluorescence Intensity (a.u.)
Wavelength (nm)

E

F

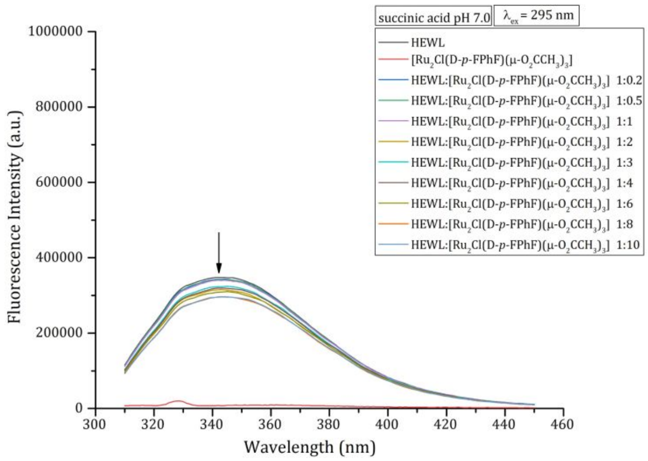
succinic acid pH 7.0
λ_{ex} = 295 nm
HEWL
$[Ru_2Cl(D\text{-}p\text{-}FPhF)(\mu\text{-}O_2CCH_3)_3]$
HEWL:$[Ru_2Cl(D\text{-}p\text{-}FPhF)(\mu\text{-}O_2CCH_3)_3]$ 1:0.2
HEWL:$[Ru_2Cl(D\text{-}p\text{-}FPhF)(\mu\text{-}O_2CCH_3)_3]$ 1:0.5
HEWL:$[Ru_2Cl(D\text{-}p\text{-}FPhF)(\mu\text{-}O_2CCH_3)_3]$ 1:1
HEWL:$[Ru_2Cl(D\text{-}p\text{-}FPhF)(\mu\text{-}O_2CCH_3)_3]$ 1:2
HEWL:$[Ru_2Cl(D\text{-}p\text{-}FPhF)(\mu\text{-}O_2CCH_3)_3]$ 1:3
HEWL:$[Ru_2Cl(D\text{-}p\text{-}FPhF)(\mu\text{-}O_2CCH_3)_3]$ 1:4
HEWL:$[Ru_2Cl(D\text{-}p\text{-}FPhF)(\mu\text{-}O_2CCH_3)_3]$ 1:6
HEWL:$[Ru_2Cl(D\text{-}p\text{-}FPhF)(\mu\text{-}O_2CCH_3)_3]$ 1:8
HEWL:$[Ru_2Cl(D\text{-}p\text{-}FPhF)(\mu\text{-}O_2CCH_3)_3]$ 1:10
Fluorescence Intensity (a.u.)
Wavelength (nm)

Figure S4. Fluorescence emission spectra of HEWL in (**A-B**) 10 mM sodium acetate buffer pH 4.0, (**C-D**) 10 mM Hepes buffer pH 7.5 and (**E-F**) 10 mM succinic acid pH 7.0 upon titration with a solution of [Ru_2Cl(D-*p*-FPhF)(O_2CCH_3)$_3$]. Spectra have been collected using λ_{exc} = 280 nm (panels **A**, **C** and **E**) and 295 nm (panels **B**, **D** and **F**).

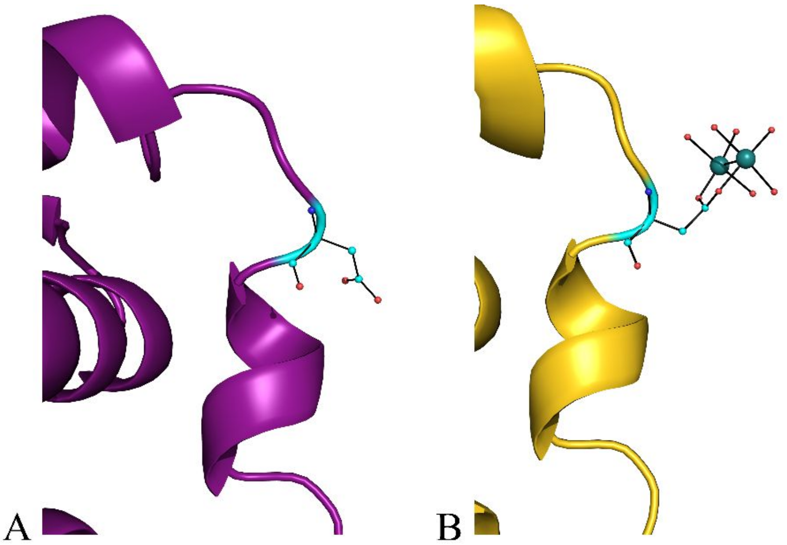

Figure S5. Conformation of Asp119 side chain in the metal-free HEWL (panel A, violet) and in structure **2** (panel B, yellow).

Table S1. Data collection and refinement statistics.

	Structure 1	Structure 2	Structure 3	Structure 4
**PDB code**	**8BPH**	**8BPU**	**8BPJ**	**8BQM**
Crystallization conditions	20% ethylene glycol, 0.1 M sodium acetate buffer at pH 4.0, 0.6 M sodium nitrate	2.0 M sodium formate, 0.1 M Hepes buffer pH 7.5	0.8 M succinic acid pH 7.0	1.1 M sodium chloride, 0.1 M sodium acetate buffer pH 4.0
Soaking time	Two weeks	Two weeks	Two weeks	Two weeks
Data collection				
Space group	$P4_3\ 2_1\ 2$	$P4_3\ 2_1\ 2$	$P4_3\ 2_1\ 2$	$P4_1\ 2_1\ 2$
a (Å)	77.48	78.36	77.19	77.74
b (Å)	77.48	78.36	77.19	77.74
c (Å)	37.27	37.51	37.83	37.38
α/β/γ (°)	90.0/90.0/90.0	90.0/90.0/90.0	90.0/90.0/90.0	90.0/90.0/90.0
Molecules for asymmetric unit	1	1	1	1
Resolution range (Å)	54.79-1.38 (1.40-1.38)	55.41-1.81 (1.84-1.81)	38.60-1.07 (1.09-1.07)	54.970- 1.17 (1.19-1.17)
Observations	244494 (11745)	228866 (10963)	949875 (16689)	852398 (19928)
Unique reflections	23817(1153)	11121 (524)	49110 (1737)	39386 (1830)
Completeness (%)	99.9 (100.0)	99.5 (98.5)	96.8 (69.7)	99.6 (95.0)
Redundancy	10.3 (10.2)	206 (20.9)	19.3 (9.6)	21.6 (10.9)
Rmerge (%)	0.047 (0.935)	0.191 (1.328)	0.042 (0.809)	0.040 (0.970)
Average I/σ(I)	21.8 (2.3)	11.2 (2.5)	33.8 (2.8)	37.8 (2.3)
$CC_{1/2}$	0.999 (0.833)	0.993 (0.874)	1.000 (0.830)	1.000 (0.740)
Anom. completeness (%)	100.0 (100.0)	99.5 (98.5)	96.6 (68.5)	99.7 (95.8)
Anom. Multiplicity	5.5 (5.3)	11.3 (11.1)	10.3 (5.1)	11.5 (5.7)
Refinement				
Resolution (Å)	1.38	1.81	1.07	1.17
N° reflections	22704	10488	46382	37067
N° reflections in working set	1638	749	2225	2603
Rfactor/Rfree	0.192/0.231	0.232/0.289	0.187/0.209	0.199/0.225
N° non-H atoms in the refin.	1257	1135	1308	1296
Ramachandran statistcs				
Most favoured	101 (95.28%)	121 (95.28%)	104 (96.30%)	97 (96.04%)
Outliers	0	0	0	0
Rmsd bonds (Å)	0.013	0.008	0.016	0.015
Rmsd angles (°)	1.921	1.557	2.065	2.093
